# Crystal structure of 4-amino-3-(3-methyl-5-phenyl-1*H*-pyrazol-1-yl)-1*H*-1,2,4-triazole-5(4*H*)-thione

**DOI:** 10.1107/S205698901500938X

**Published:** 2015-05-23

**Authors:** Joel T. Mague, Shaaban K. Mohamed, Mehmet Akkurt, Mustafa R. Albayati

**Affiliations:** a Department of Chemistry, Tulane University, New Orleans, LA 70118, USA; b Faculty of Science & Engineering, School of Healthcare Science, Manchester Metropolitan University, Manchester M1 5GD, England; c Chemistry Department, Faculty of Science, Minia University, 61519 El-Minia, Egypt; d Department of Physics, Faculty of Sciences, Erciyes University, 38039 Kayseri, Turkey; e Kirkuk University, College of Education, Department of Chemistry, Kirkuk, Iraq

**Keywords:** crystal structure, amino­triazoles, hydrogen bonding

## Abstract

In the title compound, C_12_H_12_N_6_S, the dihedral angles between the central pyrazole ring and the pendant triazole and benzene rings are 68.01 (4) and 59.83 (9)°, respectively. In the crystal, mol­ecules are linked by N—H⋯N and N—H⋯S hydrogen bonds, generating (10-1) sheets.

## Related literature   

For the bio-activities of amino­triazoles, see: Jin *et al.* (2007 ▸); Joung *et al.* (2000 ▸). For amino­triazoles as block-building synthons, see: Curtis (2004 ▸).
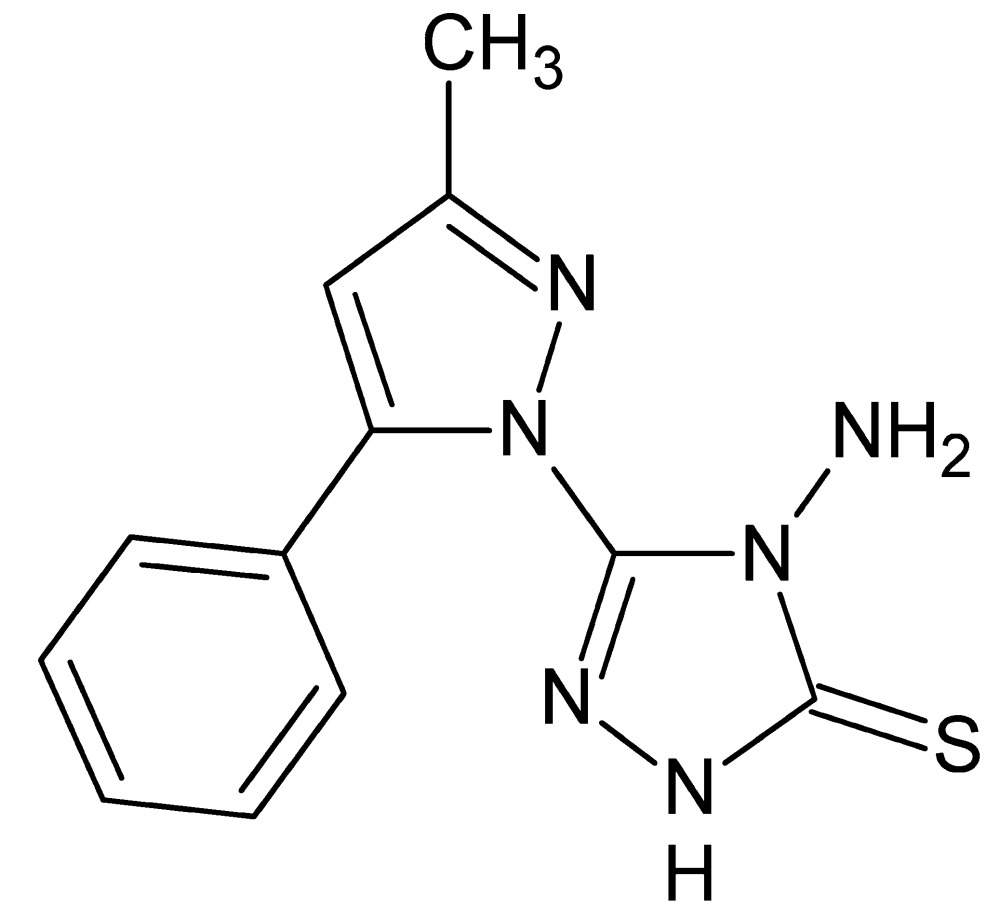


## Experimental   

### Crystal data  

C_12_H_12_N_6_S
*M* *_r_* = 272.34
Monoclinic, 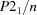

*a* = 11.3278 (4) Å
*b* = 8.3970 (3) Å
*c* = 15.4427 (5) Å
β = 109.053 (1)°
*V* = 1388.43 (8) Å^3^
*Z* = 4
Cu *K*α radiation
μ = 2.04 mm^−1^
*T* = 150 K
0.24 × 0.18 × 0.10 mm

### Data collection  

Bruker D8 VENTURE PHOTON 100 CMOS diffractometer
Absorption correction: multi-scan (*SADABS*; Bruker, 2014 ▸) *T*_min_ = 0.77, *T*_max_ = 0.82
10355 measured reflections
2683 independent reflections
2515 reflections with *I* > 2σ(*I*)
*R*_int_ = 0.021

### Refinement  

*R*[*F* ^2^ > 2σ(*F* ^2^)] = 0.036
*wR*(*F* ^2^) = 0.102
*S* = 1.09
2683 reflections
173 parameters
H-atom parameters constrained
Δρ_max_ = 0.36 e Å^−3^
Δρ_min_ = −0.28 e Å^−3^

### 

Data collection: *APEX2* (Bruker, 2014 ▸); cell refinement: *SAINT* (Bruker, 2014 ▸); data reduction: *SAINT*; program(s) used to solve structure: *SHELXT* (Sheldrick, 2015*a* ▸); program(s) used to refine structure: *SHELXL2014* (Sheldrick, 2015*b* ▸); molecular graphics: *DIAMOND* (Brandenburg & Putz, 2012 ▸); software used to prepare material for publication: *SHELXTL* (Sheldrick, 2008 ▸).

## Supplementary Material

Crystal structure: contains datablock(s) global, I. DOI: 10.1107/S205698901500938X/hb7427sup1.cif

Structure factors: contains datablock(s) I. DOI: 10.1107/S205698901500938X/hb7427Isup2.hkl

Supporting information file. DOI: 10.1107/S205698901500938X/hb7427Isup3.cml

**Fig. 1.**
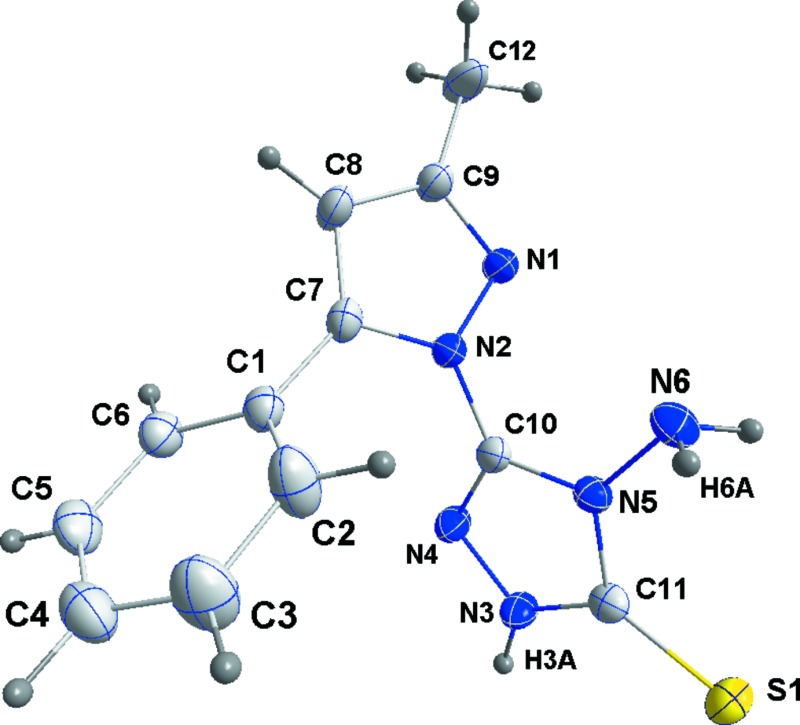
The title molecule showing labeling scheme and 50% probability ellipsoids.

**Fig. 2.**
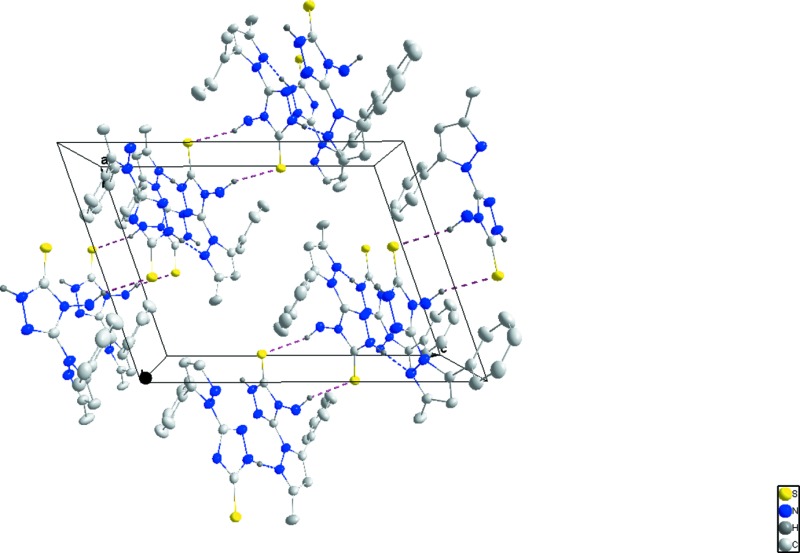
Packing viewed down the b axis. N—H···N and N—H···S hydrogen bonds are shown, respectively, as blue and purple dotted lines.

. DOI: 10.1107/S205698901500938X/hb7427fig1.tif
The title mol­ecule showing labeling scheme and 50% probability ellipsoids.

b . DOI: 10.1107/S205698901500938X/hb7427fig2.tif
Packing viewed down the *b* axis. N—H⋯N and N—H⋯S hydrogen bonds are shown, respectively, as blue and purple dotted lines.

CCDC reference: 1401505

Additional supporting information:  crystallographic information; 3D view; checkCIF report

## Figures and Tables

**Table 1:** Hydrogen-bond geometry (, )

*D*H*A*	*D*H	H*A*	*D* *A*	*D*H*A*
N3H3*A*N1^i^	0.91	1.94	2.8429(17)	169
N6H6*A*S1^ii^	0.91	2.55	3.4157(13)	159
N6H6*B*N4^iii^	0.91	2.43	3.0059(18)	122

Symmetry codes: (i) 
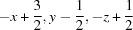
; (ii) 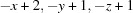; (iii) 
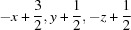
.

## supplementary crystallographic information

### S1. Comment

Amino-1,2,4-triazoles are known to be biologically active compounds (Jin *et al*., 2007). For example, the 5-amino-1,2,4- triazole itself has been used as the pesticide Amitrole (Joung *et al.*, 2000) and 3,5-diamino-1,2,4-triazole (Guanazole) is an antitumor drug that inhibits ribonucleotide reductase and DNA synthesis. In addition, they play an important role as amidine type synthons in heterocyclic chemistry (Curtis, 2004) particularly fused ring systems, such as imidazo[1,2-*b*][1,2,4]triazole, imidazo[2,1-*c*][1,2,4]triazole, 1,2,4-triazolo[1,5-*a*]pyrimidine and 1,2,4-traizolo[1,5-*a*][1,3,5]triazine possessing variety of biological effects. In this context, we report in this study the synthesis and crystal structure of the title compound.

In the title compound (Fig. 1), the dihedral angle between the central 5-membered ring and its attached phenyl ring is 59.83 (5)° while the dihedral angle between the two 5-membered rings is 68.01 (4)°. In the crystal, the molecules form sheets lying parallel to (101) through N—H···N and N—H···S hydrogen bonds (Fig. 2 and Table 1).

### S2. Experimental

A mixture of 1 mmol (258 mg) of 5-(3-methyl-5-phenyl-1*H*-pyrazol-1-yl)-1,3,4-oxadiazole-2(3*H*)-thione and 2 ml of hydrazine in 30 ml ethanol was heated at 351 K for 6 h. On cooling, the solid product was filtered off, dried under vacuum and recrystallized from ethanol to afford colorless blocks of the title compound.

### S3. Refinement

H-atoms attached to carbon were placed in calculated positions (C—H = 0.95 - 0.98 Å) while those attached to nitrogen were placed in locations derived from a difference map and their parameters adjusted to give N—H = 0.91 Å. All were included as riding contributions with isotropic displacement parameters 1.2 - 1.5 times those of the attached atoms.

### Crystal data

**Table:**

C_12_H_12_N_6_S	*F*(000) = 568
*M**_r_* = 272.34	*D*_x_ = 1.303 Mg m^−^^3^
Monoclinic, *P*2_1_/*n*	Cu *K*α radiation, λ = 1.54178 Å
*a* = 11.3278 (4) Å	Cell parameters from 8668 reflections
*b* = 8.3970 (3) Å	θ = 4.3–72.3°
*c* = 15.4427 (5) Å	µ = 2.04 mm^−^^1^
β = 109.053 (1)°	*T* = 150 K
*V* = 1388.43 (8) Å^3^	Block, colourless
*Z* = 4	0.24 × 0.18 × 0.10 mm

### Data collection

**Table:**

Bruker D8 VENTURE PHOTON 100 CMOS diffractometer	2683 independent reflections
Radiation source: INCOATEC IµS micro-focus source	2515 reflections with *I* > 2σ(*I*)
Mirror monochromator	*R*_int_ = 0.021
Detector resolution: 10.4167 pixels mm^-1^	θ_max_ = 72.3°, θ_min_ = 4.2°
ω scans	*h* = −13→13
Absorption correction: multi-scan (*SADABS*; Bruker, 2014)	*k* = −9→10
*T*_min_ = 0.77, *T*_max_ = 0.82	*l* = −19→19
10355 measured reflections	

### Refinement

**Table:**

Refinement on *F*^2^	Primary atom site location: structure-invariant direct methods
Least-squares matrix: full	Secondary atom site location: difference Fourier map
*R*[*F*^2^ > 2σ(*F*^2^)] = 0.036	Hydrogen site location: mixed
*wR*(*F*^2^) = 0.102	H-atom parameters constrained
*S* = 1.09	*w* = 1/[σ^2^(*F*_o_^2^) + (0.0581*P*)^2^ + 0.5187*P*] where *P* = (*F*_o_^2^ + 2*F*_c_^2^)/3
2683 reflections	(Δ/σ)_max_ = 0.002
173 parameters	Δρ_max_ = 0.36 e Å^−^^3^
0 restraints	Δρ_min_ = −0.28 e Å^−^^3^

### Special details

**Table:**

Geometry. All e.s.d.'s (except the e.s.d. in the dihedral angle between two l.s. planes)are estimated using the full covariance matrix. The cell e.s.d.'s are takeninto account individually in the estimation of e.s.d.'s in distances, anglesand torsion angles; correlations between e.s.d.'s in cell parameters are onlyused when they are defined by crystal symmetry. An approximate (isotropic)treatment of cell e.s.d.'s is used for estimating e.s.d.'s involving l.s. planes.
Refinement. Refinement of *F*^2^ against ALL reflections. The weighted *R*-factor *wR* andgoodness of fit *S* are based on *F*^2^, conventional *R*-factors *R* are basedon *F*, with *F* set to zero for negative *F*^2^. The threshold expression of*F*^2^ > σ(*F*^2^) is used only for calculating *R*-factors(gt) *etc*. and isnot relevant to the choice of reflections for refinement. *R*-factors basedon *F*^2^ are statistically about twice as large as those based on *F*, and *R*-factors based on ALL data will be even larger. H-atoms attached to carbonwere placed in calculated positions (C—H = 0.95 - 0.98 Å) while thoseattached to nitrogen were placed in locations derived from a differencemap and their parameters adjusted to give N—H = 0.91 Å. All wereincluded as riding contributions with isotropic displacementparameters 1.2 - 1.5 times those of the attached atoms.

### Fractional atomic coordinates and isotropic or equivalent isotropic displacement parameters (Å^2^)

**Table:**

	*x*	*y*	*z*	*U*_iso_*/*U*_eq_	
S1	1.06212 (3)	0.49379 (4)	0.36297 (2)	0.02864 (14)	
N1	0.52909 (11)	0.54447 (14)	0.29113 (8)	0.0249 (3)	
N2	0.60148 (11)	0.41615 (13)	0.33081 (8)	0.0235 (3)	
N3	0.86566 (11)	0.29657 (15)	0.28254 (8)	0.0263 (3)	
H3A	0.9039	0.2256	0.2559	0.032*	
N4	0.74091 (11)	0.27516 (15)	0.27154 (9)	0.0273 (3)	
N5	0.81912 (11)	0.48358 (13)	0.35855 (8)	0.0205 (3)	
N6	0.81859 (11)	0.61690 (14)	0.41236 (8)	0.0262 (3)	
H6A	0.8704	0.5948	0.4699	0.031*	
H6B	0.8512	0.6973	0.3878	0.031*	
C1	0.60875 (14)	0.17810 (17)	0.42641 (9)	0.0251 (3)	
C2	0.72215 (17)	0.1972 (2)	0.49645 (11)	0.0399 (4)	
H2	0.7559	0.3009	0.5120	0.048*	
C3	0.78631 (19)	0.0666 (2)	0.54370 (13)	0.0469 (5)	
H3	0.8636	0.0807	0.5914	0.056*	
C4	0.73738 (18)	−0.0845 (2)	0.52122 (12)	0.0392 (4)	
H4	0.7808	−0.1744	0.5538	0.047*	
C5	0.62567 (16)	−0.10472 (18)	0.45161 (11)	0.0344 (4)	
H5	0.5927	−0.2088	0.4362	0.041*	
C6	0.56078 (15)	0.02533 (18)	0.40384 (11)	0.0292 (3)	
H6	0.4839	0.0102	0.3559	0.035*	
C7	0.54349 (13)	0.31865 (17)	0.37531 (9)	0.0239 (3)	
C8	0.43020 (14)	0.38690 (19)	0.36366 (10)	0.0287 (3)	
H8	0.3670	0.3482	0.3862	0.034*	
C9	0.42509 (14)	0.52643 (18)	0.31143 (10)	0.0262 (3)	
C10	0.71675 (13)	0.39120 (16)	0.31857 (9)	0.0218 (3)	
C11	0.91672 (13)	0.42285 (16)	0.33429 (9)	0.0222 (3)	
C12	0.32313 (15)	0.6470 (2)	0.28071 (13)	0.0386 (4)	
H12A	0.3481	0.7316	0.2466	0.058*	
H12B	0.3074	0.6928	0.3343	0.058*	
H12C	0.2469	0.5957	0.2411	0.058*	

### Atomic displacement parameters (Å^2^)

**Table:**

	*U*^11^	*U*^22^	*U*^33^	*U*^12^	*U*^13^	*U*^23^
S1	0.0210 (2)	0.0357 (2)	0.0290 (2)	−0.00344 (13)	0.00779 (15)	−0.00294 (13)
N1	0.0233 (6)	0.0232 (6)	0.0304 (6)	0.0020 (5)	0.0120 (5)	0.0040 (5)
N2	0.0235 (6)	0.0206 (6)	0.0301 (6)	0.0012 (4)	0.0136 (5)	0.0031 (4)
N3	0.0235 (6)	0.0263 (6)	0.0330 (6)	−0.0013 (5)	0.0145 (5)	−0.0075 (5)
N4	0.0242 (6)	0.0270 (6)	0.0340 (6)	−0.0030 (5)	0.0141 (5)	−0.0049 (5)
N5	0.0224 (6)	0.0193 (5)	0.0210 (5)	0.0013 (4)	0.0088 (5)	−0.0009 (4)
N6	0.0311 (7)	0.0225 (6)	0.0248 (6)	0.0018 (5)	0.0089 (5)	−0.0048 (4)
C1	0.0288 (7)	0.0241 (7)	0.0251 (7)	−0.0021 (5)	0.0125 (6)	0.0010 (5)
C2	0.0459 (10)	0.0302 (8)	0.0355 (8)	−0.0082 (7)	0.0019 (7)	0.0026 (6)
C3	0.0468 (11)	0.0443 (10)	0.0382 (9)	−0.0008 (8)	−0.0018 (8)	0.0085 (8)
C4	0.0500 (10)	0.0327 (9)	0.0369 (8)	0.0094 (7)	0.0168 (7)	0.0099 (6)
C5	0.0477 (10)	0.0223 (7)	0.0372 (8)	0.0004 (6)	0.0195 (7)	−0.0018 (6)
C6	0.0316 (8)	0.0262 (7)	0.0313 (8)	−0.0027 (6)	0.0122 (6)	−0.0033 (6)
C7	0.0258 (7)	0.0230 (7)	0.0242 (6)	−0.0061 (5)	0.0101 (5)	−0.0012 (5)
C8	0.0246 (7)	0.0313 (8)	0.0333 (7)	−0.0045 (6)	0.0136 (6)	0.0045 (6)
C9	0.0223 (7)	0.0283 (7)	0.0294 (7)	−0.0019 (5)	0.0104 (6)	0.0014 (6)
C10	0.0217 (7)	0.0208 (6)	0.0242 (6)	0.0001 (5)	0.0094 (5)	0.0018 (5)
C11	0.0244 (7)	0.0229 (7)	0.0207 (6)	0.0014 (5)	0.0091 (5)	0.0014 (5)
C12	0.0262 (8)	0.0422 (9)	0.0521 (10)	0.0079 (7)	0.0193 (7)	0.0143 (8)

### Geometric parameters (Å, º)

**Table:**

S1—C11	1.6697 (14)	C2—C3	1.384 (2)
N1—C9	1.3230 (19)	C2—H2	0.9500
N1—N2	1.3714 (16)	C3—C4	1.383 (3)
N2—C7	1.3670 (18)	C3—H3	0.9500
N2—C10	1.3949 (18)	C4—C5	1.377 (3)
N3—C11	1.3401 (18)	C4—H4	0.9500
N3—N4	1.3790 (17)	C5—C6	1.387 (2)
N3—H3A	0.9100	C5—H5	0.9500
N4—C10	1.2968 (18)	C6—H6	0.9500
N5—C10	1.3639 (18)	C7—C8	1.363 (2)
N5—C11	1.3759 (17)	C8—C9	1.413 (2)
N5—N6	1.3953 (15)	C8—H8	0.9500
N6—H6A	0.9101	C9—C12	1.492 (2)
N6—H6B	0.9099	C12—H12A	0.9800
C1—C2	1.392 (2)	C12—H12B	0.9800
C1—C6	1.392 (2)	C12—H12C	0.9800
C1—C7	1.477 (2)		
			
C9—N1—N2	104.65 (11)	C4—C5—H5	119.6
C7—N2—N1	112.32 (11)	C6—C5—H5	119.6
C7—N2—C10	127.07 (12)	C5—C6—C1	119.77 (15)
N1—N2—C10	120.47 (11)	C5—C6—H6	120.1
C11—N3—N4	113.70 (11)	C1—C6—H6	120.1
C11—N3—H3A	127.7	C8—C7—N2	105.56 (12)
N4—N3—H3A	118.6	C8—C7—C1	133.80 (13)
C10—N4—N3	103.20 (11)	N2—C7—C1	120.55 (12)
C10—N5—C11	107.82 (11)	C7—C8—C9	106.58 (12)
C10—N5—N6	123.89 (11)	C7—C8—H8	126.7
C11—N5—N6	128.28 (12)	C9—C8—H8	126.7
N5—N6—H6A	107.0	N1—C9—C8	110.89 (13)
N5—N6—H6B	105.4	N1—C9—C12	120.25 (13)
H6A—N6—H6B	109.8	C8—C9—C12	128.85 (14)
C2—C1—C6	119.08 (14)	N4—C10—N5	112.16 (12)
C2—C1—C7	119.82 (13)	N4—C10—N2	124.53 (13)
C6—C1—C7	121.07 (13)	N5—C10—N2	123.26 (12)
C3—C2—C1	120.73 (16)	N3—C11—N5	103.12 (11)
C3—C2—H2	119.6	N3—C11—S1	129.55 (11)
C1—C2—H2	119.6	N5—C11—S1	127.32 (11)
C4—C3—C2	119.73 (16)	C9—C12—H12A	109.5
C4—C3—H3	120.1	C9—C12—H12B	109.5
C2—C3—H3	120.1	H12A—C12—H12B	109.5
C5—C4—C3	119.98 (16)	C9—C12—H12C	109.5
C5—C4—H4	120.0	H12A—C12—H12C	109.5
C3—C4—H4	120.0	H12B—C12—H12C	109.5
C4—C5—C6	120.70 (15)		
			
C9—N1—N2—C7	0.12 (16)	N2—N1—C9—C8	−0.21 (16)
C9—N1—N2—C10	176.19 (12)	N2—N1—C9—C12	178.88 (14)
C11—N3—N4—C10	−0.45 (16)	C7—C8—C9—N1	0.23 (18)
C6—C1—C2—C3	−0.5 (3)	C7—C8—C9—C12	−178.76 (16)
C7—C1—C2—C3	−178.43 (16)	N3—N4—C10—N5	−0.20 (15)
C1—C2—C3—C4	0.0 (3)	N3—N4—C10—N2	−177.56 (12)
C2—C3—C4—C5	0.5 (3)	C11—N5—C10—N4	0.75 (15)
C3—C4—C5—C6	−0.4 (3)	N6—N5—C10—N4	179.53 (12)
C4—C5—C6—C1	−0.1 (2)	C11—N5—C10—N2	178.15 (12)
C2—C1—C6—C5	0.5 (2)	N6—N5—C10—N2	−3.1 (2)
C7—C1—C6—C5	178.45 (13)	C7—N2—C10—N4	64.2 (2)
N1—N2—C7—C8	0.02 (16)	N1—N2—C10—N4	−111.26 (16)
C10—N2—C7—C8	−175.74 (13)	C7—N2—C10—N5	−112.89 (16)
N1—N2—C7—C1	−176.94 (12)	N1—N2—C10—N5	71.66 (18)
C10—N2—C7—C1	7.3 (2)	N4—N3—C11—N5	0.88 (15)
C2—C1—C7—C8	−118.65 (19)	N4—N3—C11—S1	−178.60 (11)
C6—C1—C7—C8	63.4 (2)	C10—N5—C11—N3	−0.94 (14)
C2—C1—C7—N2	57.3 (2)	N6—N5—C11—N3	−179.65 (12)
C6—C1—C7—N2	−120.64 (15)	C10—N5—C11—S1	178.55 (10)
N2—C7—C8—C9	−0.14 (16)	N6—N5—C11—S1	−0.2 (2)
C1—C7—C8—C9	176.23 (15)		

### Hydrogen-bond geometry (Å, º)

**Table:**

*D*—H···*A*	*D*—H	H···*A*	*D*···*A*	*D*—H···*A*
N3—H3*A*···N1^i^	0.91	1.94	2.8429 (17)	169
N6—H6*A*···S1^ii^	0.91	2.55	3.4157 (13)	159
N6—H6*B*···N4^iii^	0.91	2.43	3.0059 (18)	122

Symmetry codes: (i) −*x*+3/2, *y*−1/2, −*z*+1/2; (ii) −*x*+2, −*y*+1, −*z*+1; (iii) −*x*+3/2, *y*+1/2, −*z*+1/2.
