# Supplementary figures and images for: Crystal structure of 4-amino-3-(3-methyl-5-phenyl-1H-pyrazol-1-yl)-1H-1,2,4-triazole-5(4H)-thione
Source: Acta Crystallogr E Crystallogr Commun. 2015 May 23;71(Pt 6):o417. doi: 10.1107/S205698901500938X (PMC4459321; doi:10.1107/S205698901500938X)

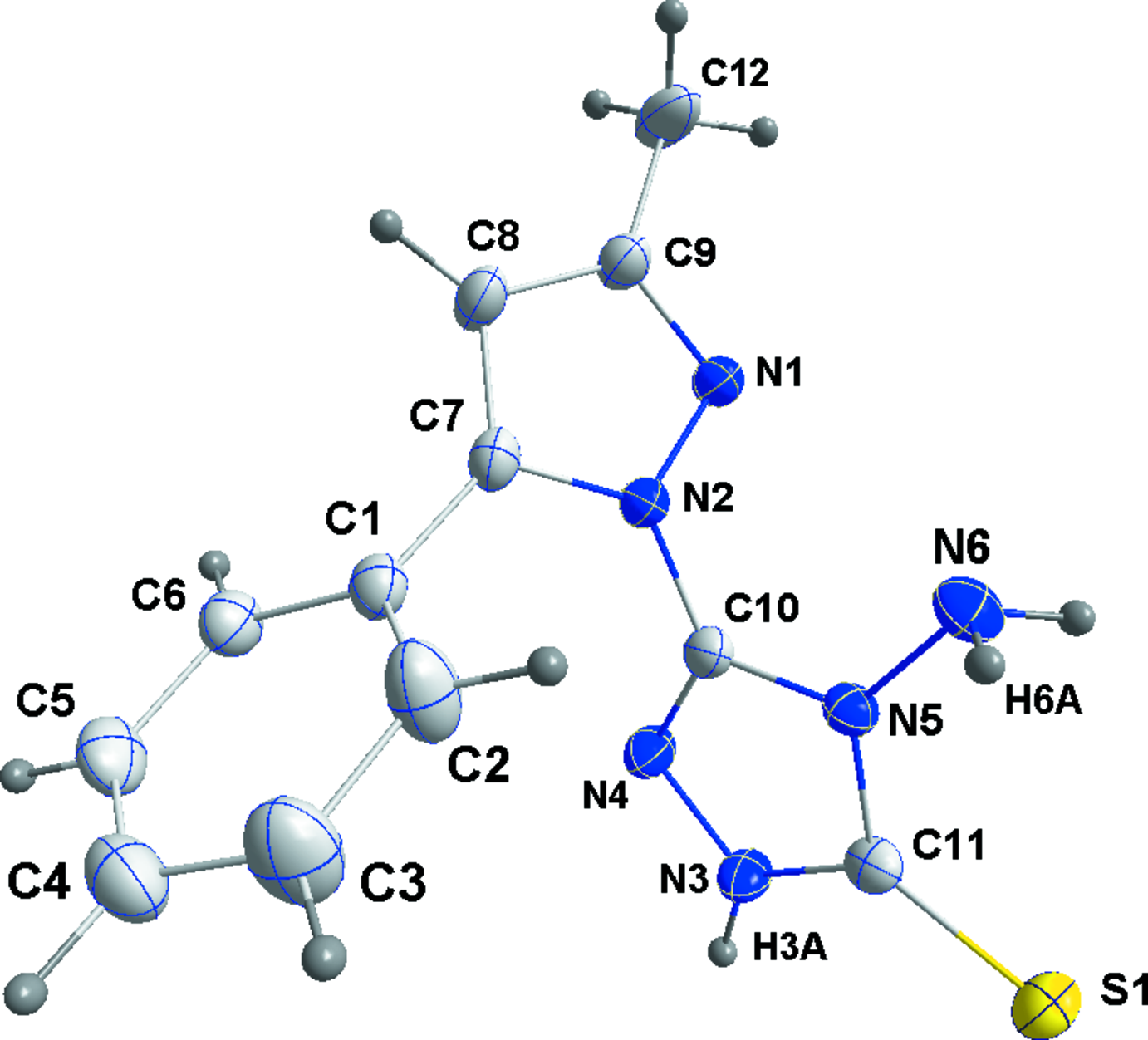

Supplement: Supplementary file 4 [file e-71-0o417-fig1.tif]

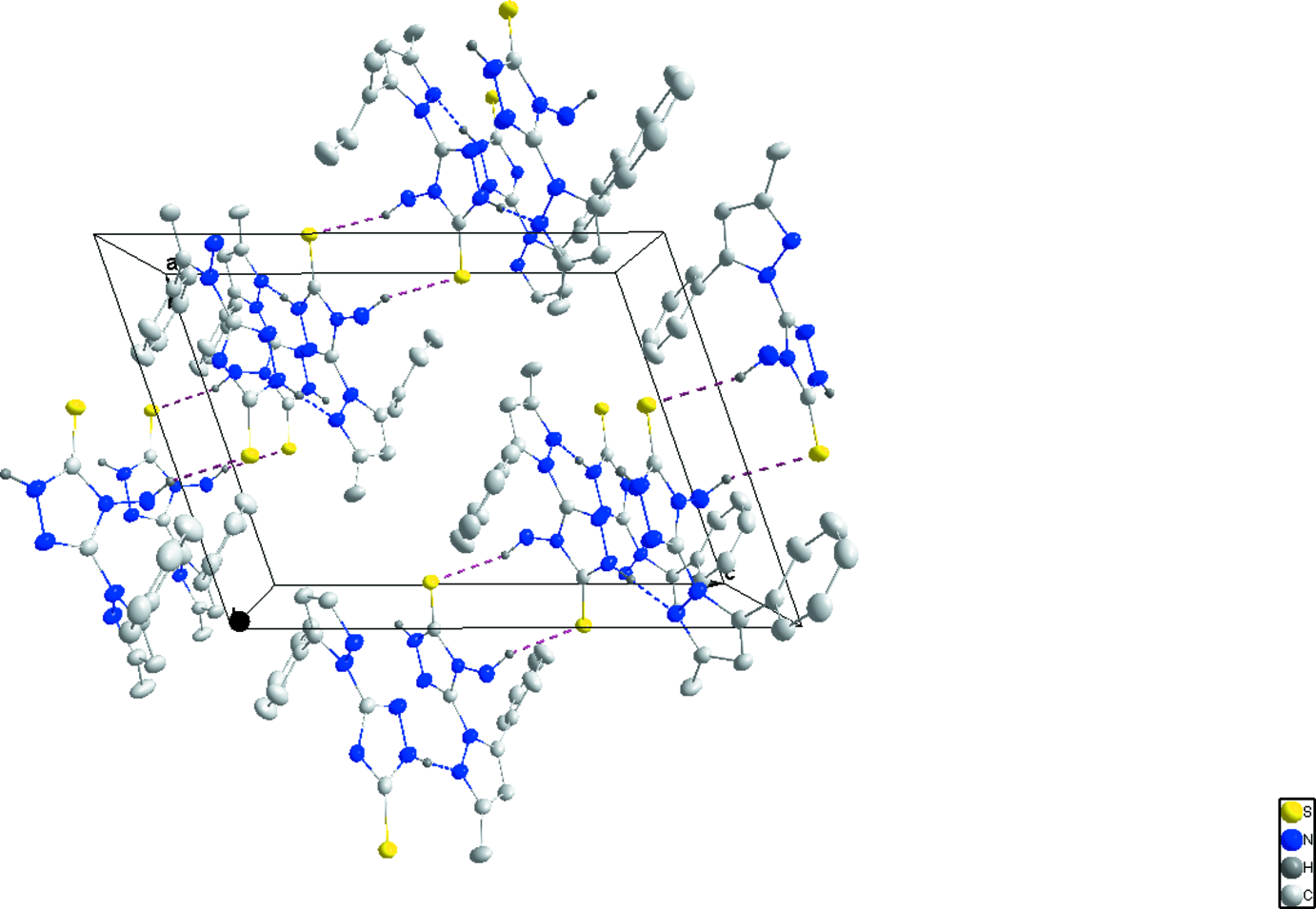

Supplement: Supplementary file 5 [file e-71-0o417-fig2.tif]
